# The missing middle of childhood

**DOI:** 10.1080/16549716.2023.2242196

**Published:** 2023-08-07

**Authors:** Maj-Lis Voss, Mariam Claeson, Sven Bremberg, Stefan Swartling Peterson, Tobias Alfvén, Grace Ndeezi

**Affiliations:** a NorthSouth Group for Poverty Reduction, Moss Beach, CA, USA; b Political Economy of Adolescent Mental Health, Department of Global Public Health, Karolinska Institute, Stockholm, Sweden; c Global Public Health, Department of Global Public Health, Karolinska Institute, Stockholm, Sweden; d Department of Global Public Health, Karolinska Institute, Stockholm, Sweden; e Department of Pediatrics and Child Health, Makerere University, Kampala, Uganda

**Keywords:** Missing middle, childhood, outcomes, risk, indicators, standardization

## Abstract

Middle childhood, between six and twelve years, is a critical bridge between earlier childhood and adolescence with rapid physical and psychological transitions. Most of the world’s 2.6 billion young people, of which the middle childhood age group is a significant portion, live in low- and middle-income countries. Many live in environments that place them at high and growing risk for mental ill-health, injuries, and adoption of risky behaviours that often lead to non-communicable diseases in later years. Still, middle childhood, the ‘missing middle,’ is omitted from global health information systems, targeted policies, and strategies. The dearth of internationally comparable and standardised indicators on middle childhood in major international development agency databases hampers age-appropriate policy and programme development. Better understanding of the needs of this increasingly vulnerable population is critical. Middle childhood needs to be an explicit focus within child-focused research and implementation. Standardised, comprehensive, and relevant indicators are required to quantify the contribution of middle childhood to the global burden of disease and to facilitate interventions, monitoring, and evaluation, to ensure that all children flourish and thrive.

## The missing middle

Of the world’s 2.6 billion people below the age of nineteen, 940 million are in the six- to twelve-year age range [1]. This transitional period from childhood to adolescence, middle childhood, is a distinct and pivotal developmental stage with rapid physical and psychological transitions that influence outcomes throughout the life-course. In middle childhood, children experience a slowdown in physical growth and begin to refine their fine motor skills, learn about social relationships, and rely on their ability to regulate their emotions. The ability for planning and self-direction is evident in middle childhood. As children transition into adolescence, they face a period of dramatic change with physical growth spurts and sexual maturation, and abstract concepts such as love and freedom take hold in this developmental stage [2]. This transitional period of middle childhood remains missing from almost all global health information systems as a unique population group and is rarely the focus of health policies and strategies. Risky practices and exposure to harm begin earlier in a young person’s life today, compared to a generation ago. Many opportunities are missed to educate, nurture, and help all children in this period of childhood thrive. Displaced children are extremely vulnerable, at risk for poverty and starvation, violence and crime, and sexual and labour exploitation. The number of child migrants increased from 24 million to 36 million between 1990 and 2000 [3]. The child refugee population more than doubled between 2001 and 2021, from 4 million to over 10 million, outnumbering adult refugees [4]. The use of new and rapidly evolving technology is a double-edged sword, where children in some areas do not have access to advances in information technology as a result of the digital divide, while children and adolescents with access to social media, are at risk for crime, bullying, exploitation, and unhealthy marketing efforts. The introduction of smartphones around 2011 brought about a generational shift globally where children and adolescent users have been able to live their entire lives connected to social networking sites and the influence they exert [5]. Millions of children are left without digital access, however, according to UNICEF: it is estimated that about two-thirds of the world’s school children have no internet connection at home [6].

Different life experiences, cultural and social norms, and environments in which children live contribute to outcomes in childhood and beyond, and investments made in health and education, protection and prevention, empowerment, and well-being during childhood have the potential to greatly improve outcomes throughout the life-course [7]. Poor knowledge about the transition from early childhood to adolescence, the ‘missing middle’, hampers national and international efforts to identify and explicitly address the specific risks of children 6–12 years old,^1^^1^Lower and upper age ranges vary between early, middle, and late childhood across data sources as discussed in this paper. Without standardized age ranges in the child growth and development literature, we have chosen the age span six to twelve years for middle childhood for the purpose of this discussion paper. especially among those living in environments with disproportionately higher risks for violence, injury, mental ill-health, and adoption of risky behaviours, including practices that drive overweight and obesity.

In contrast, a significant amount of attention has been given to early childhood. Global attention to children under the age of five, focusing on child survival and early childhood development is reflected in comprehensive research and in a wealth of data available from a wide range of sources. More recent attention to adolescence, including regional variations in pattern of morbidity and mortality caused by injury, non-communicable diseases, reproductive health, and communicable diseases [8] has led to the development of indicators to track progress on adolescent health and well-being that provide a better understanding about this often-tumultuous time.

## Global indicators on child and adolescent health and well-being

Age ranges in global child health databases are non-standardised and do not include the age segment 6–12 as a population of interest. Although health practitioners agree that this age group and their needs are qualitatively different from younger children and adolescents, the lack of comparable data hampers the design of policy and practice to ensure that all children flourish throughout childhood and adolescence [9].

Although WHO has recently recognised the ‘missing middle’ as a distinct period of growth and development [10], this age group continues to be subsumed within broader labels. Similarly, UNICEF and World Bank data contain a range of age sub-groups, excluding middle childhood as a unique population [11,12]. The age ranges are not standardised and vary widely, making specific comparisons uncertain (i.e. 0–4, 0–5, 0–8, 0–14, 0–19, 5–9, 5–19, 5–24, 6–10, 7–14, 10–14, 10–19, 11–17, 13–15, and 15–19). Moreover, the Sustainable Development Goals (SDGs) aim in its Goal 3 to ‘ensure healthy lives and promote well-being for all at all ages’ and to put children’s thriving and flourishing at the top of the development agenda’. Indicators target children in only two age groups, however: children under age five and adolescents 15–19 years old, missing the middle [13].

Social structures for delineating ages six to twelve as a unique population have in recent years become less definite, but we know that middle childhood is a period of rapid shifts in cognition, motivation, and social behaviour during which early interventions can have significant impact on long-term outcomes [14]. Efforts to target specific vulnerabilities in middle childhood are impeded by the non-standardised age groupings in global health information systems. Children today live with a multitude of challenges relating to physical and mental health. Sleep disorders, asthma, and cancers are growing problems, and obesity and mental ill-health are seen in increasingly younger children. According to a recent multi-agency report, food insecurity and hunger have increased, and malnutrition continues to be a major public health problem, putting children at risk for developmental delays. In 2022 among children under five years of age, an estimated 148 million were stunted and 45 million were wasted. Similar data on middle childhood is not available [15]. This brief overview focuses on two large and growing challenges to children’s health and well-being of particular importance during middle childhood: mental ill-health and obesity.

## Mental ill-health

Children and adolescents worldwide are increasingly vulnerable due to climate change, ecological degradation, forced migration, inequities, injury and violence, unhealthy lifestyles, predatory commercial marketing, and harmful digital technology [16]. Across income levels and geographies, increased globalisation has brought about a convergence of threats to children’s physical and mental health. For example, almost 90% of the world’s children live without clean air. Ten to twenty per cent of children and adolescents worldwide suffer from mental ill-health [17]. Anxiety disorders appear to peak at around age ten [18], and body dissatisfaction between the ages 6 and 10. The pressure to be thin primarily among girls, ‘appearance culture’, is most prevalent in the 9-to-12 age group [19]. Behavioural disorders, ADHD, bullying and problems with friends, and peer group pressures commonly appear before age 12, as does conduct disorder (more prevalent among boys) [20]. More than 100 countries, mostly low- and middle-income countries, lack comparable data on child and adolescent mental health status [21]. While data on access to children’s mental health services are hard to come by, UNICEF reports in its State of the World’s Children 2021 that ‘the number of psychiatrists specialising in treating children and adolescents was fewer that 0.1% per 100,000 in all but high-income countries where the figure was 5.5% per 100,000’. The resulting economic and social costs of poor mental health in childhood and adolescence are not yet fully understood but are of great concern, with suicide being the third leading cause of death in the 15-to-19 year old population. Yet, most countries allocate less than two per cent of their budgets to mental health, a figure that has remained unchanged for many years [22].

COVID-19 related disruptions to education, livelihoods, and social connections have added stressors that disproportionately impact children’s physical and mental health, particularly in low resource settings. The negative effects of COVID-19 on children and young people include increased rates of depression, fear and anxiety, loss of social connections, and exposure to domestic violence. The more severe long-term negative impact may be that of a reversal in the progress made pre-pandemic in the areas of child health, education, and protection [23].

We know that information technology plays an increasingly powerful role in shaping self-image, values, and perceptions among children. Global media, anecdotal evidence, and non-technical literature indicate a host of negative self- and body-image issues affecting increasingly younger children as they transition to adolescence. It is also known that rates of depression, self-harm, and anxiety increased significantly around the time that digital media became a daily part of children’s lives [24]. Children are exposed to online bullying and abuse, and contact with criminals and sexual predators, but the impacts are not yet fully understood due to lack of data [25].

## Overweight and obesity

Another non-communicable and related condition of major global public health concern is overweight and obesity among children. While the number of underweight children worldwide between the ages 5–19 continues to be larger than those who are overweight or obese, in 2020, 150 million were overweight or obese. These numbers are estimated to reach 40 and 254 million, respectively, by 2030 [26]. Low- and middle-income countries are seeing a rapid increase in overweight and obese children: in Africa, the number of overweight children under-5 has increased by 24% since 2000; and in 2019, almost half of the children under-5 who were overweight or obese lived in Asia. Significant variations exist regionally and between and within countries, however. Between 1975 and 2016, for example, obesity among those 5–19 years old in high-income countries increased by 30–50% per decade while in Southern Africa each decade saw a 400% increase [27]. In the Asia-Pacific region, obesity rates among primary school children in rural Bangladesh were estimated at 3.5% in 2018, around 12% in China, and 30% in Iran and Saudi Arabia [28]. The 20/21 New Zealand Health Survey found that almost 13% of children aged 2–14 were obese. Between 1990 and 2011, the prevalence of overweight and obesity in school-aged students in the Eastern Mediterranean Region ranged from 70% to 45% [29]. The global economic burden of high Body Mass Index (BMI) is estimated to cost health services US $990 billion per year, representing 13% of all healthcare expenditures [30]. By the year 2025, one-third of the global population will be overweight or obese, increasing the number of non-communicable disease-related deaths and putting SDG 3 by 2030 beyond reach [31]. In low resource settings, health services may not be able to bear the obesity-related costs and the result could be a significant fall in life expectancy [32,33].

## Measuring trends in middle childhood

While international development agency databases such as WHO, UNICEF and the World Bank offer national, regional, and global estimates of child health indicators, most focus on mortality and a limited number of conditions. Lack of comprehensive, relevant, and widely available core data on the large middle childhood population entering adolescence hinders our understanding of risks and challenges. Mortality-focused indicators alone are misleading measures of child and adolescent health [34] and omit a multitude of issues likely to negatively impact prospects for health and well-being throughout the life-course. Notable exceptions exist in which six- to twelve-year-old children are included as part of other age groups and can be analysed as an age group of interest in terms of physical and mental health [35], including The Children’s Worlds project which focuses on children’s lives around the world from their own perspective, a much-needed area of study [36], and the Measuring Mental Health Outcomes among Adolescents and Youth at the Population level (MMAP) which enables youth engagement in local and cultural adaptation of survey instruments, and could be used to measure these indices in middle childhood [37].

Given the importance of schools during middle childhood, school-based interventions have been adopted in many countries at national, regional, and local levels to strengthen capacity to respond to the challenges faced by children and their caregivers, parents, teachers, and communities. UNICEF and WHO-supported school-based programmes to promote mental and psycho-social well-being include Communities That Care, Strong Minds, and Helping Adolescents Thrive [38]. In 2022, UNESCO, UNICEF, and WHO called on governments to take action that includes school-based interventions towards promoting child and adolescent well-being so that every child can thrive [39]. Conducting global school-based self-reported surveys as a first step would provide an understanding of the overall well-being and subsequent needed interventions in middle childhood. While much debate surrounds the scientific reliability and validity of self-reporting among young children, it would, nevertheless, provide an indication of health status as the child himself/herself perceives it. Carrying out such surveys requires an understanding of the relationship between culture and health, that is, cultural competency and cultural humility, particularly in cross-cultural environments [40]. As part of MMAP, UNICEF has developed a culturally adaptable tool, a global set of indicators in an effort to address cultural diversity towards strengthening our understanding of adolescent mental health [41].

We reviewed eleven major peer-reviewed journals, and PubMed, on health, global health, epidemiology, and health economics to assess trends in research between the year 2001 and 2020 relating to the stage between childhood and adolescence. An online search was conducted on the number of journal article titles containing the terms childhood, middle childhood, preadolescence, and adolescence. Results (Table 1) show a striking lack of research on middle childhood and preadolescence: of 1183 articles published in the selected journals none focused on middle childhood or preadolescence. Similarly, of the nearly 74,000 articles on childhood, middle childhood, preadolescence, and adolescence housed in PubMed during the same time period, less than 2% focused on middle childhood and preadolescence.^2^^2^Search results are based on the number of journal article titles that include the terms childhood; middle childhood; preadolescence; or adolescence..

**Table 1.** Number of published articles with search terms: childhood, middle childhood, preadolescence, and adolescence, in periods 2001–2010 and 2011–2020.

		Search terms			
Journal	Date range	Childhood	Middle childhood	Preadolescence	Adolescence
BMJ Global Health (est 2016)	2001–2010	NA	NA	NA	NA
	2011–2020	22	0	0	0
Global Public Health	2001–2010	1	0	0	2
	2011–2020	5	0	0	0
Global Health Research and Policy (est 2016)	2001–2010	NA	NA	NA	NA
	2011–2020	2	0	0	0
International Health (est 2009)	2001–2010	NA	NA	NA	NA
	2011–2020	0	0	0	1
International Journal of Epidemiology	2001–2010	52	0	0	8
	2011–2020	86	0	0	13
International Journal of Public Health	2001–2010	3	0	0	2
	2011–2020	22	0	0	7
Journal of Epidemiology and Global Health (est 2015)	2001–2010	NA	NA	NA	NA
	2011–2020	0	0	0	0
Journal of Health Economics	2001–2010	0	0	0	0
	2011–2020	1	0	0	1
The Lancet Child and Adolescent Health (est 2017)	2001–2010	NA	NA	NA	NA
	2011–2020	398	0	0	165
The Lancet Global Health (est 2013)	2001–2010	NA	NA	NA	NA
	2011–2020	315	0	0	36
WHO Bulletin	2001–2010	27	0	0	0
	2011–2020	14	0	0	0
National Library of Medicine (PubMed)	2001–2010	22,271	220	29	3,505
	2011–2020	38,588	903	115	8,021

## In conclusion

Risky practices and exposure to harm begin early in a young person’s life today, requiring new approaches and strategies across sectors. Today’s children and adolescents face growing challenges and threats: injuries, violence, substance abuse, unsafe sex, mental ill-health, and obesity. While these are mostly preventable, the rapidly changing socio-economic, political, demographic, and technological realities are likely to further increase vulnerabilities.

Reducing the burden of major diseases such as mental ill-health and obesity in middle childhood requires data, policies and earlier interventions that recognise the particular needs of this population. Universal acceptance of age group standards establishing a specific ‘middle childhood’ category will facilitate cross-country comparisons and estimation of middle childhood contribution to the global burden of disease.

Country-level technical capacity needs to be strengthened, and targeted and culturally adapted indicators with age-appropriate communications tools and survey instruments need to be developed to complement current generic ones. At the global level, coordinated integrated multi-sector interventions are critical for the collection, management, and dissemination of such data and to inform decision makers at global and national levels.

Middle childhood is a unique developmental stage with identifiable vulnerabilities that are overlooked in current health information systems and global development goals. This critical age range is an optimal window of opportunity to achieve positive outcomes and close the equity gap related to future health, educational attainment, and social and emotional functioning. Better age-appropriate strategies focusing on children between early childhood and adolescence are essential in helping this ‘missing middle’ to thrive and flourish.

## Paper context

Middle childhood is left out of global development goals, health information systems and policies and programmes. The ‘missing middle’ (children between the ages six-to-twelve) is exposed to risks and challenges that often result in poor mental health, overweight and obesity, including adverse outcomes later in life. Middle childhood is an optimal time in the life-course to promote health and wellbeing, but comprehensive and standardised data, age-appropriate policies, and programmes are lacking.
